# The Histone Demethylase PHF8 Is Essential for Endothelial Cell Migration

**DOI:** 10.1371/journal.pone.0146645

**Published:** 2016-01-11

**Authors:** Lunda Gu, Juliane Hitzel, Franziska Moll, Christoph Kruse, Randa Abdel Malik, Jens Preussner, Mario Looso, Matthias S. Leisegang, Dieter Steinhilber, Ralf P. Brandes, Christian Fork

**Affiliations:** 1 Institute for Cardiovascular Physiology, Medical Faculty, Goethe-University Frankfurt, Germany; 2 Institute of Vascular Signalling, Centre for Molecular Medicine, Goethe-University Frankfurt, Germany; 3 Max Planck Institute for Heart and Lung Research, Bad Nauheim, Germany; 4 Institute of Pharmaceutical Chemistry/ZAFES, Goethe-University Frankfurt, Frankfurt am Main, Germany; 5 German Center for Cardiovascular Research (DZHK), Partner site RheinMain, Frankfurt, Germany; The Walter and Eliza Hall of Medical Research, AUSTRALIA

## Abstract

Epigenetic marks critically control gene expression and thus the cellular activity state. The functions of many epigenetic modifiers in the vascular system have not yet been studied. We screened for histone modifiers in endothelial cells and observed a fairly high expression of the histone plant homeodomain finger protein 8 (PHF8). Given its high expression, we hypothesize that this histone demethylase is important for endothelial cell function. Overexpression of PHF8 catalyzed the removal of methyl-groups from histone 3 lysine 9 (H3K9) and H4K20, whereas knockdown of the enzyme increased H3K9 methylation. Knockdown of PHF8 by RNAi also attenuated endothelial proliferation and survival. As a functional readout endothelial migration and tube formation was studied. PHF8 siRNA attenuated the capacity for migration and developing of capillary-like structures. Given the impact of PHF8 on cell cycle genes, endothelial E2F transcription factors were screened, which led to the identification of the gene repressor E2F4 to be controlled by PHF8. Importantly, PHF8 maintains E2F4 but not E2F1 expression in endothelial cells. Consistently, chromatin immunoprecipitation revealed that PHF8 reduces the H3K9me2 level at the E2F4 transcriptional start site, demonstrating a direct function of PHF8 in endothelial E2F4 gene regulation. Conclusion: PHF8 by controlling E2F4 expression maintains endothelial function.

## Introduction

An intact endothelial barrier is essential for vascular function. It prevents vessel occlusion and controls vascular permeability. After vascular injury, endothelial cells locally proliferate, enlarge and migrate to restore an intact vascular surface. Indeed, endothelial cell death (apoptosis), dysfunction or senescence has been implicated in the pathogenesis of numerous vascular diseases such as atherosclerosis, thrombosis and vascular leakage [1–3].

Epigenetic control of gene expression by histone modification is a central mechanism determining cell-fate and cell-phenotype maintenance [4]. Compared to the generally permissive histone modification through acetylation, histone methylations are more diverse in function and their regulation is complex and dynamic. Methylated histones are associated with promoter activation (H3 lysine 4 tri-methyl), enhancer activity (H3K4me1) and a repressive heterochromatin structure (H3K9me2/3, K27me2/3) [5,6]. Enzymes responsible for these modifications are histone methyltransferases and demethylases. The epigenetic control by those enzymes, however, is incompletely understood. Given the great importance of endothelial cells for vascular biology, fairly little is known about the function of histone methylation modifying enzymes and first publications are just emerging [7–9].

In this study we focused on the enzyme plant homeodomain finger protein 8 (PHF8). The biology of this histone demethylase is inadequately understood but its relevance for one human disease has already been demonstrated: Mutations of PHF8 are a cause for the X-linked intellectual disability but vascular phenotypes have not been reported [10,11]. The enzymatic function of PHF8 is probably to demethylate H3K9, H3K27 and H4K20 [12–17]. Through this mechanism, the enzyme is thought to regulate key cellular processes like ribosomal RNA transcription, notch signaling and cytoskeleton dynamics [12,18,19]. In zebrafish, it could be shown that PHF8 regulates brain and craniofacial development but vascular defects were not reported [13].

As we observed significant mRNA expression of PHF8 in endothelial cells and based on its broad impact on gene regulation, we hypothesize that PHF8 also impacts on endothelial cell function.

## Materials and Methods

### Materials

Human recombinant TNFα (#300-01A) was purchased from PeproTech (Rocky Hill, NY, USA), cycloheximide from Sigma-Aldrich (München, Germany). Anti-PHF8 (#ab36068), anti-H4K20me1 (#ab9051) and anti-H4 (#ab10158) were purchased from abcam. The second anti-PHF8 was from bethyl (#A301-772A). Anti-H3K9me1 (#pAB065-050), anit-H3K9me2 (#C15410060), anit-H3K27me1 (#pAb-045-050), anti-H3K27me2 (#pAb-046-050), H3K4me3 (#pAb-003-050) and anti-H3 (#C15200011) were from diagenode. Topoisomerase 1 (#sc5342) and anti-Tubulin beta (#sc-9104) antibodies were acquired from Santa Cruz (Heidelberg, Germany). Anti-ßActin (#A1978) was purchased from Sigma-Aldrich (München, Germany).

### Cell culture

Human umbilical vein endothelial cells (HUVECs) were purchased from Lonza (#CC-2519, Lot No.186864; 191772; 192485; 76524; 76921, 7F3111, Walkersville, MD, USA) and PELOBiotech (#PB-CH-190-8013, Lot No. QC-18P13F11, Planegg, Germany). Cells were cultured on fibronectin-coated (#356009, Corning Incorporated, Tewksbury, MA USA) dishes in endothelial growth medium (EGM), consisting of endothelial basal medium (EBM) supplemented with human recombinant epidermal growth factor (EGF), EndoCGS-Heparin, (PELOBiotech, Planegg, Germany), 8% fetal calf serum (FCS) (#S0113, Biochrom, Berlin, Germany), penicillin (50 U/ml) and streptomycin (50 μg/ml) (#15140–122, Gibco (lifetechnologies, Carlsbad, CA, USA) in a humidified atmosphere of 5% CO2 at 37°C. For each experiment at least three different batches of HUVEC from passage 3 were used.

Immortalized human microvascular endothelial cells (HMEC-1) were provided by CDC (#98247 Atlanta, GA, USA). Cells were cultured on fibronectin-coated dishes in endothelial growth medium (EGM). For experiments cells from passage 6 were used.

Human embryonic kidney (HEK) 293T/17 cells (#CRL-11268) were purchased from ATCC (Manassas, VA, USA). Cells were cultured in Dulbecco's Modified Eagle's Medium (DMEM), high glucose, GlutaMAX from Gibco, Lifetechnologies (Carlsbad, CA, USA), supplemented with 8% fetal calf serum (FCS), penicillin (50 U/ml), and streptomycin (50 μg/ml) in a humidified atmosphere of 5% CO2 at 37°C.

Human aortic smooth muscle cells (HAoSMC)(#354-05a) were purchased from PELOBiotech (Planegg, Germany). Cells were cultured in Smooth Muscle Cell Medium (#PB-MH-200-2190) supplemented with 8% fetal calf serum (FCS), penicillin (50 U/ml), streptomycin (50 μg/ml), EGF, FGF, glutamin and insulin from singlequots (PELOBiotech, Planegg, Germany). Cells were cultured in a humidified atmosphere of 5% CO2 at 37°C.

Human foreskin fibroblasts were cultured in DMEM/F12 (#11039–021) from Gibco (Lifetechnologies, Carlsbad, CA, USA) supplemented with 8% fetal calf serum (FCS), penicillin (50 U/ml), and streptomycin (50 μg/ml) in a humidified atmosphere of 5% CO2 at 37°C.

### shRNA, siRNA and plasmid transfection

For shRNA treatment, endothelial cells were infected with lentiviral particles according to Addgene “plKO.1 Protocol” (http://www.addgene.org/tools/protocols/plko/). Cells were selected with puromycin (0.5 μg/ml). The PHF8 target sequence was: 5’- CCGGCCCAACTGTGAAGTCTTGCATCTCGAGATGCAAGACTTCACAGTTGGGTTTTTG -3’. Control shRNA against green fluorescent protein (shGFP) and shScrambled (shScr) were purchased from Addgene. For siRNA treatment, endothelial cells (80–90% confluent) were transfected with GeneTrans II according to the instructions provided by MoBiTec (Göttingen,Germany). siRNAs for PHF8 were purchased from Sigma (siPHF8-1: #SASI_Hs01_00079031, siPHF8-2: #SASI_Hs01_00079033, siPHF8-3, SASI_Hs01_00079032). siE2F4 was purchased from ThermoFisher Scientific (#114193 siE2F4-1 and #114194 siE2F4-2). Control siRNAs (siScrambled) were from Invitrogen (siScr-1: #12935–300, siScr-2: #12935112, siScr-3: #12935113). Plasmid overexpression was achieved with the Neon electroporation system (Invitrogen). The plasmid PHF8 (vector: pcDNA3) was kindly provided by Shi Yang (Harvard Medical School, Department of Cell Biology) [13], E2F1 and E2F4 (vector: pcDNA3) plasmids were from Addgene (24225# and #10914).

### Quantitative RT-PCR

Total RNA was extracted with the RNA Mini Kit (Bio&Sell). cDNA was prepared with SuperScript III reverse transcriptase (Invitrogen) and random hexamer together with oligo(dT) primers (Sigma #O4387). Quantitative real-time PCR was performed with Eva Green Master Mix and ROX as reference dye (Bio&Sell #76.580.5000) in a Mx3005 cycler (Stratagene). Relative expression of target genes were normalized to (RNA)II (DNA-directed) polypeptide A (POLR2A) or ß-Actin and analyzed by the delta-delta Ct method with the MxPro software (Agilent Technologies, Santa Clara, CA, USA).

### Protein isolation and western blot analysis

Cells were lysed with Triton X-100 lysis buffer (20 mM TRIS/Cl pH 7.5, 150 mM NaCl, 10 mM NaPPi, 20 mM NaF, 1% Triton, 2 mM orthovanadate (OV), 10 nM okadaic Acid, protein-inhibitor mix (PIM), 40 μg/ml phenylmethylsulfonylfluorid). Cells were centrifuged for 10 min at 16,000 g. Supernatant and pellet were used and after determination of protein concentration by the Bradford assay, equal amounts of proteins were boiled in Laemmli buffer and separated by SDS-PAGE gel electrophoresis. Infrared-fluorescent-dye-conjugated secondary antibodies were purchased from Licor (Bad Homburg, Germany) and detected with an infrared-based laser scanning detection system (Odyssey Classic, Licor, Bad Homburg, Germany). For nucleus protein extraction cells were lysed in a buffer containing (10 mM HEPES pH 7.9, 10 mM KCl, 0.1 mM EDTA, 0.1 mM EGTA, 10 mM DTT, PIM and PMSF). After incubation on ice for 15 min, Nonidet was added (0.75%) and cells were centrifuged for 1 min at 16,000 g. Pellet was lysed in a buffer (20 mM Tris-HCL pH 7.5, 300 mM NaCl, 0.5% Triton X-100, 1% SDS, 1 mM DTT, PMSF, PIM) containing 0.033 u Benzonase (Sigma) and 2 u DNase (Promega) and rotated with 8 rpm by 4°C for 30–60 min. Subsequently, proteins were boiled in Laemmli buffer.

### Chromatin immuno-precipitation (ChIP)

Cell preparation (2x10^6^ HUVECs), crosslinking and nuclei isolation were performed with the truCHIP™ Chromatin Shearing Kit (Covaris, Woburn, USA) according to the manufacturers protocol. Afterwards, the lysates were sonified with the Bioruptur Plus (9 cycles, 30 seconds on, 90 seconds off; Diagenode, Seraing, Belgium) at 4°C. Cell debris were removed by centrifugation and the lysates were diluted 1:3 in dilution buffer (20 mmol/L Tris/HCl pH 7.4, 100 mmol/L NaCl, 2 mmol/L EDTA, 0.5% Triton X-100 and protease inhibitors). After preclearing with 20 μL DiaMag protein A coated magnetic beads (Diagenode, Seraing, Belgium) for 30 minutes at 4°C, samples were incubated over night at 4°C with 3 μg of anti-H3K9me1, anit-H3K9me2, anti-H4K20me1 and anti-H4 (diagenode #pAB065-050, # diagenode C15410060, # abcam ab9051 # abcam ab10158). 5% of the samples served as input. The antibody complexes were collected with 35 μL DiaMag protein A coated magnetic beads (Diagenode, Seraing, Belgium) for 3 hours at 4°C, subsequently washed twice for 5 minutes with each of the wash buffers 1–3 (Wash Buffer 1: 20 mmol/L Tris/HCl pH 7.4, 150 mmol/L NaCl, 0.1% SDS, 2 mmol/L EDTA, 1% Triton X-100; Wash Buffer 2: 20 mmol/L Tris/HCl pH 7.4, 500 mmol/L NaCl, 2 mmol/L EDTA, 1% Triton X-100; Wash Buffer 3: 10 mmol/L Tris/HCl pH 7.4, 250 mmol/L lithium chloride, 1% Nonidet, 1% sodium deoxycholate, 1 mmol/L EDTA) and finally washed with TE-buffer pH 8.0. Elution of the beads was done with elution buffer (0.1 M NaHCO3, 1% SDS) containing 1x proteinase K (Diagenode, Seraing, Belgium) and shaking at 600 rpm for 1 hour at 55°C, 1 hour at 62°C and 10 minutes at 95°C. After removal of the beads, the eluate was purified with the QiaQuick PCR purification kit (Qiagen, Hilden, Germany) and subjected to qPCR analysis. Chip data were calculated relative to input.

### Cell migration

Scratch wound-healing assays were performed in 24-well plates. 1.5 x 10^5^ Cells were cultured in endothelial basal medium (EBM) containing FCS (8%). Endothelial cell migration was monitored by live cell imaging (Zeiss TIRF System LASOS77). The distance of migration was calculated using ImageJ software.

Endothelial cell migration in Boyden chamber assays were investigated in a modified transwell chamber system. 2x10^5^ cells were seeded on membrane inserts (FluoroBlok, 3 μm pore size, BD Bioscience, Heidelberg, Germany) in the presence of EBM. The lower chamber contained EBM supplemented with 1% FCS. After 20 hours, the cells on the upper surface of the filter were removed mechanically. Then cells that had migrated into the lower compartment were fixed (4% paraformaldehyde in PBS), stained with DAPI and counted.

### Tube formation

For the investigation of endothelial cells to form capillary-like structures, a tube formation assay was performed. Matrigel Growth Factor Reduced (BD) was prepared and incubated with 75x10^4^ HUVECs in EBM + 1%FCS for 7 hours. Subsequently fixed with 4% paraformaldehyde and images were acquired on an Axiovert135 microscope (Zeiss). Cumulative tube length was analyzed with the AxioVision software (Zeiss).

### Apoptosis/survival assay

Measurements were performed with 1x10^6^ HUVECs according to the protocol from chemometec “Annexin V Assay” (application note No. 3017 rev. 1.4). NucleoCounter NC-3000 (chemometec) was used for quantification.

### Propidium iodide staining

HUVECs were fixed with -20°C 70% ethanol and 5x10^5^ cells were resolved in 200 μl propidium iodide solution (50 μg/ml, 537060, Calbiochem) with 50 μl RNase (50 μg/ml) and quantified by flow cytometry. Cells were analyzed by the FACS Calibur machine (BD Biosciences, Heidelberg, Germany).

### Carboxyfluorescein succinimidyl ester (CFSE) staining.

Cell suspension (5-10x10^6^/ml) was treated with 0.5 μl 10 mM CFSE solution (#65–0850, eBioscience, affymetrix) and incubated for 10 min placed in the dark. Cells were washed 3 times with 4–5 volumes of ice cold Dulbecco's Modified Eagle's Medium with 8% fetal calf serum. Cells were analyzed by the FACS Calibur machine (BD Biosciences, Heidelberg, Germany).

### Statistics

Unless otherwise indicated, data are given as means ± standard error of mean (SEM). Calculations were performed with Graphpad PRISM 5.0. In case of multiple testing, Bonferroni correction was applied. For multiple group comparisons ANOVA followed by post hoc with Fisher LSD test was performed. Individual statistics of samples were performed by T-test. P values of <0.05 was considered as significant. Unless otherwise indicated, N indicates the number of individual experiments.

## Results

### PHF8 is expressed in human endothelial cells

**Fig 1:**
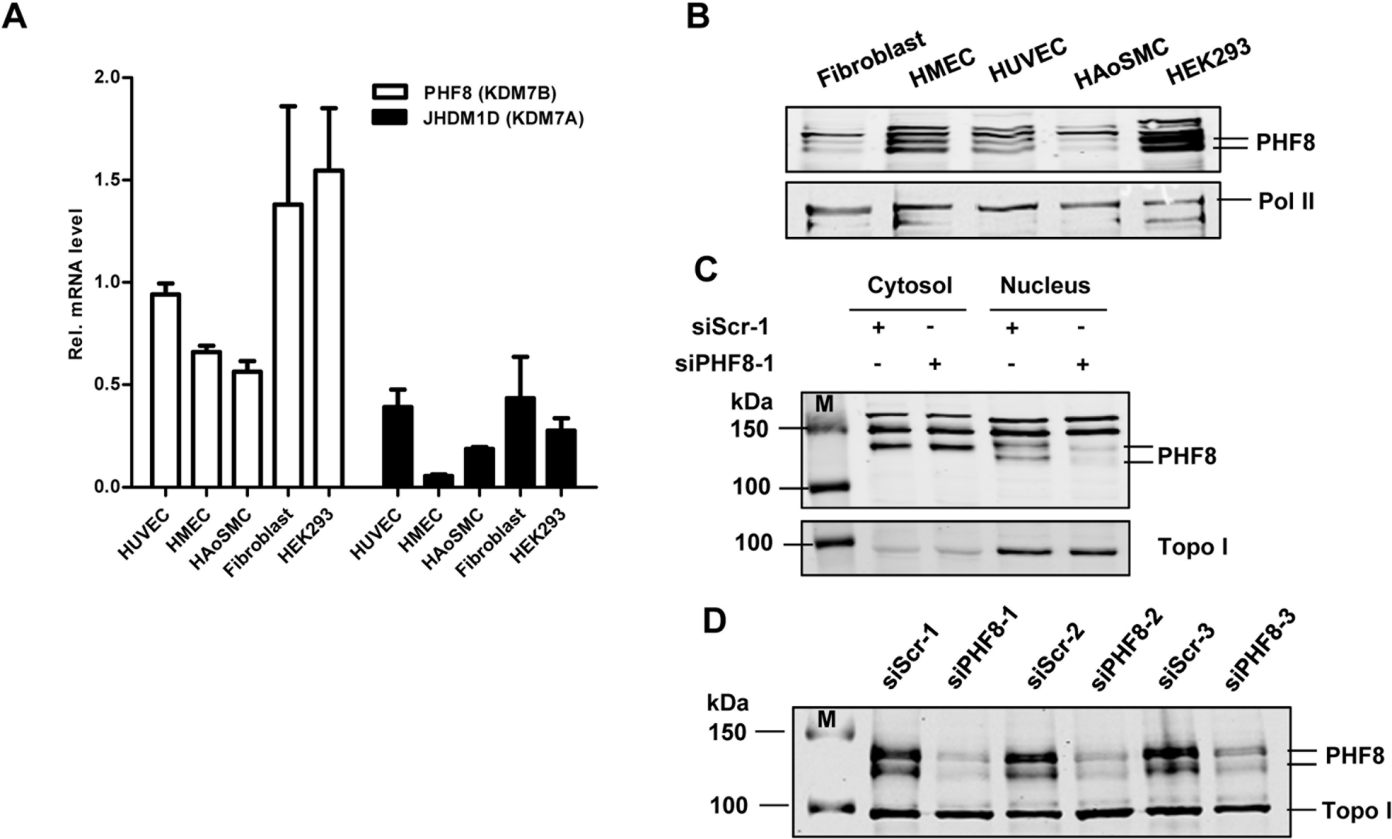
PHF8 is expressed in endothelial cells. A: Expression profile of the KDM7 family members PHF8 and JHDM1D as determined by qRT-PCR normalized to β-Actin, n = 3. B: Representative western blot of PHF8 and RNA-polymerase II (Pol II) from the nucleus of human umbilical vein endothelial cell (HUVEC), human microvascular endothelial cell line (HMEC), human aortic smooth muscle cells (HAoSMC), fibroblast and HEK293. C&D: Western blot from the cytosol and nuclear fraction (C) or only nuclear fraction (D) of HUVECs stained with anti-PHF8 (C: abcam #ab36068, D: bethyl #A301-772A) and transfected with siRNA against PHF8 or scrambled as control M = Protein Ladder.

To uncover a potential function of PHF8 in the endothelium, we first analyzed its expression in different cell types. qRT-PCR revealed that the expression of PHF8 mRNA in human umbilical vein endothelial cells (HUVECs) and microvascular endothelial cells (HMECs) is higher than that of the closest family member JHDM1D **(Fig 1A)**. Western blot analysis confirmed the expression of PHF8 protein in HUVECs and HMECs **(Fig 1B)**. A comparison between the cytoplasmic and nuclear cellular fraction revealed that the expression of PHF8 is restricted to the nucleus, which is suggestive for a potential function as histone demethylase in endothelial cells **(Fig 1C)**. As the antibody used (abcam #ab36068) exhibited significant unspecific reactivity in the cytosolic fraction as well as in the nucleus, the signal was validated by siRNA technique. Whereas the signal in the cytosolic fraction was siRNA-resistant, two of the numerous bands obtained in the nuclear fraction were attenuated by siRNA. This result could be validated with a more specific antibody (bethyl #A301-772A). With this antibody, only two bands were detected in the nuclear fraction which both decreased in response to siRNA (**Fig 1D**). Whether the two bands represent splice variants or post translational modifications of the protein is unclear.

### PHF8 decreases global levels of H3K9me1/2 and H4K20me1 in endothelial cells

**Fig 2:**
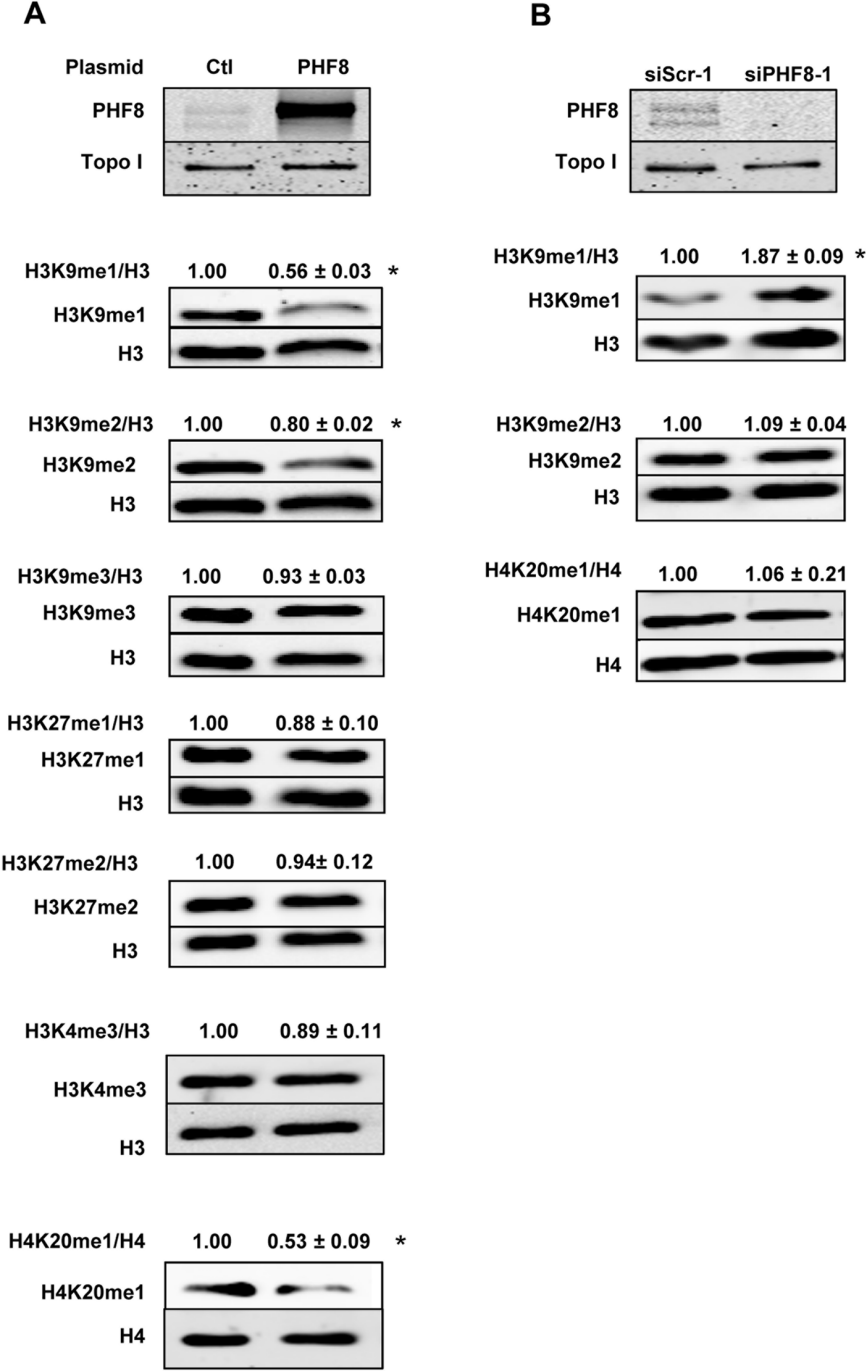
PHF8 demethylates H3K9me1/2 and H4K20me1 in endothelial cells. A: Representative western blot and densitometry for histones and their modifications as indicated from HUVECs with overexpression (A) or knockdown (B) of PHF8 by RNAi. Anti-PHF8 from bethyl was used. Scr = scrambled RNAi, Ctl = pcDNA3-GFP. Numbers above the blots indicate the results of the relative densitometry. n = 3, *p<0.05.

PHF8 has been described to demethylate several lysine residues in histone 3 and 4, such as H3K9, H3K27, and H4K20 [12–17] However, its substrate in endothelial cells is unknown. In order to address this, the enzyme was transiently overexpressed in HUVECs. Western blot analysis for histone methylation marks revealed that PHF8 overexpression reduced global levels of H3K9me1/2 and H4K20me1, whereas H3K27me1/2 and H3K4me3, the latter being thought to act as anchor for PHF8 were not affected **(Fig 2A)**. Importantly, reduction of PHF8 expression by RNA interference in HUVECs resulted in a global increase of H3K9me1. In contrast to the overexpression studies, however, this approach did not change global H3K9me2 and H4K20me1 levels **(Fig 2B).** Thus, PHF8 decreases global level of H3K9me1 and if overexpressed also of H3K9me2 and H4K20me1 in endothelial cells.

### PHF8 is required for endothelial proliferation and survival

**Fig 3:**
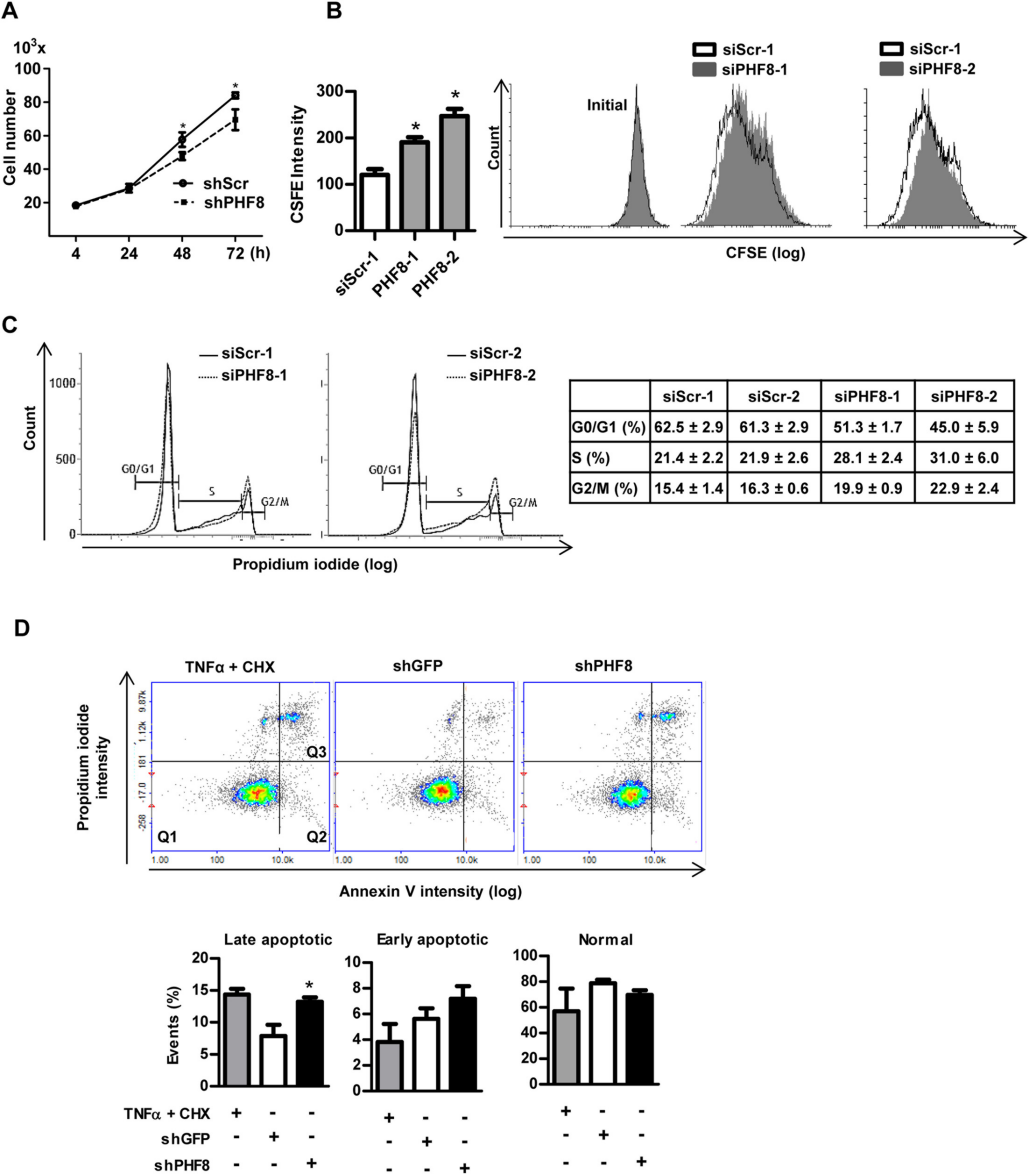
PHF8 is required for endothelial proliferation and survival. A: Proliferation analysis determined by cell counting of HUVECs transduced with control shRNA shScrambled (shScr) or shRNA against PHF8 (shPHF8), n = 5. B&C: Flow cytometry analysis of HUVEC proliferation with the CFSE dye (B) or cell cycle analysis (C) with propidium iodide after 72 h RNAi transfection, n = 5. D: Apoptosis/survival assay for late apoptosis (Q3: annexin V and propidium iodide positive cells), early apoptosis (Q2: only annexin V positive) and annexin V/propidium iodide negative cells (Q1: normal). Tumor necrosis factor alpha (TNFα, 20 ng/ml, 3h) and cycloheximide (CHX, 25 μg/ml, 3h) served as positive control. HUVECs were transduced with control shRNA (shGFP) or shPHF8, n = 3, *p<0.05.

To identify a functional role for PHF8 in endothelial cells, the effect of depletion of the enzyme on endothelial proliferation and survival was determined. Total cell number increased significantly slower in PHF8 shRNA treated HUVEC as compared to scrambled shRNA cells (**Fig 3A**). In line with this, flow cytometry analyses with the CFSE dye revealed a reduced dilution of CFSE which is suggestive for a reduced cell division rate **(Fig 3B)**. Nuclear propidium iodide staining for cell cycle analysis demonstrated that the knockdown of PHF8 led to accelerated G1/S transition but to reduced progression of S- and G2/M phase **(Fig 3C & Table 1).**

**Table 1:** Primer sequences.

Gene	Forward	Reverse
PHF8	TGCTGACATTGACCTCTACC	TTCCAGTGGGCTTCAGAATC
JHDM1D	ACCTGAATGGAGAGCGAAAG	TCATGTTCCACTCCCTCTAC
E2F1	GGACTCTTCGGAGAACTTTCAG	TGATGGTGGTGGTGACACTATG
E2F4	AGCCCATCTGCTGTTTCTAC	TCACCACTGTCCTTGCTATC
Pol II	GCACCACGTCCAATGACAT	GTGCGGCTGCTTCCATAA
ß-Actin	AAAGACCTGTACGCCAACAC	GTCATACTCCTGCTTGCTGAT

A decreased PHF8 expression, however, not only altered proliferation, it also affected cell death as observed by Annexin V / PI staining: A comparison to cells treated with the combination of TNFα and cycloheximide as positive control revealed a strong increase in late as well as early apoptotic cells in response to PHF8 depletion in HUVECs **(Fig 3D)**. Thus, PHF8 facilitates endothelial cell proliferation and survival.

### PHF8 regulates E2F4 expression in endothelial cells

**Fig 4:**
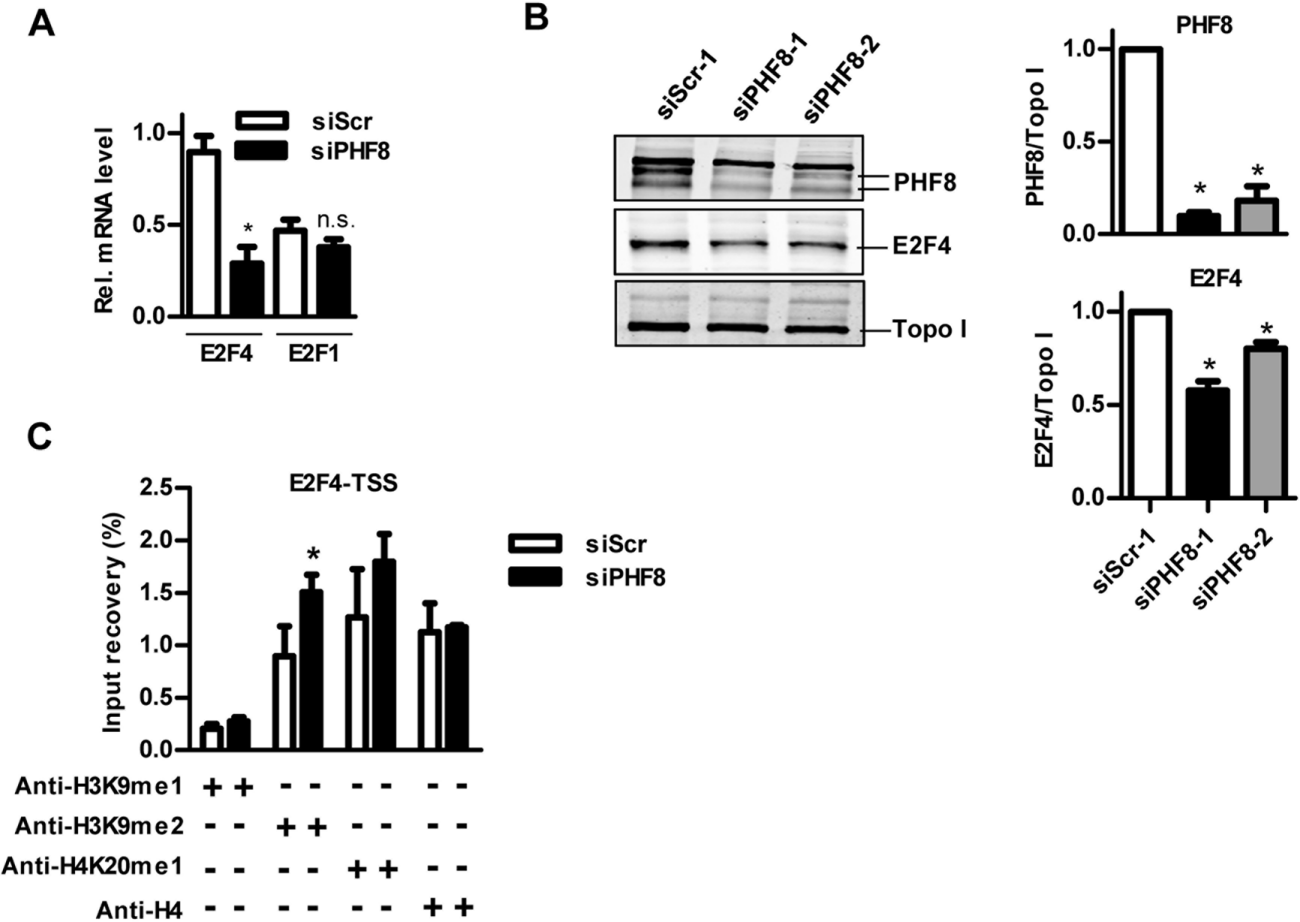
PHF8 regulates E2F4 expression in endothelial cells. A&B: qRT-PCR of E2F4, E2F1 (relative to Pol II) and representative western blot and densitometry (B) from HUVECs transfected with control siRNA (siScr) or PHF8 siRNA (siPHF8). Anti-PHF8 from abcam was used. n = 5. C: Chromatin immunoprecipitation of HUVECs transfected with control siScr or siPHF8 with the antibodies indicated followed by qPCR for E2F4 using primers binding at the transcription start site (TSS). n = 3, *p<0.05.

E2F transcription factors are involved in cell cycle regulation and have pro- (E2F1) as well as anti-apoptotic (E2F4) functions [20–23]. As PHF8 has been reported to interact with E2F1 in HeLa cells [16], the role of PHF8 for these proteins was determined in HUVECs. PHF8 siRNA significantly reduced the expression of E2F4 but not E2F1 **(Fig 4A)**. This effect was not restricted to mRNA, also E2F4 protein expression was attenuated in response to PHF8 siRNA (**Fig 4B)**. In order to relate this effect to altered histone modifications, chromatin immunoprecipitation assays were performed for the transcription start site of E2F4. The depletion of PHF8 indeed resulted in a significant enrichment of the repressive H3K9me2 mark whereas H4K20me1 or H3K9me1 signature were not affected **(Fig 4C)**.

### PHF8 maintains endothelial migration in an E2F4-dependent manner

**Fig 5:**
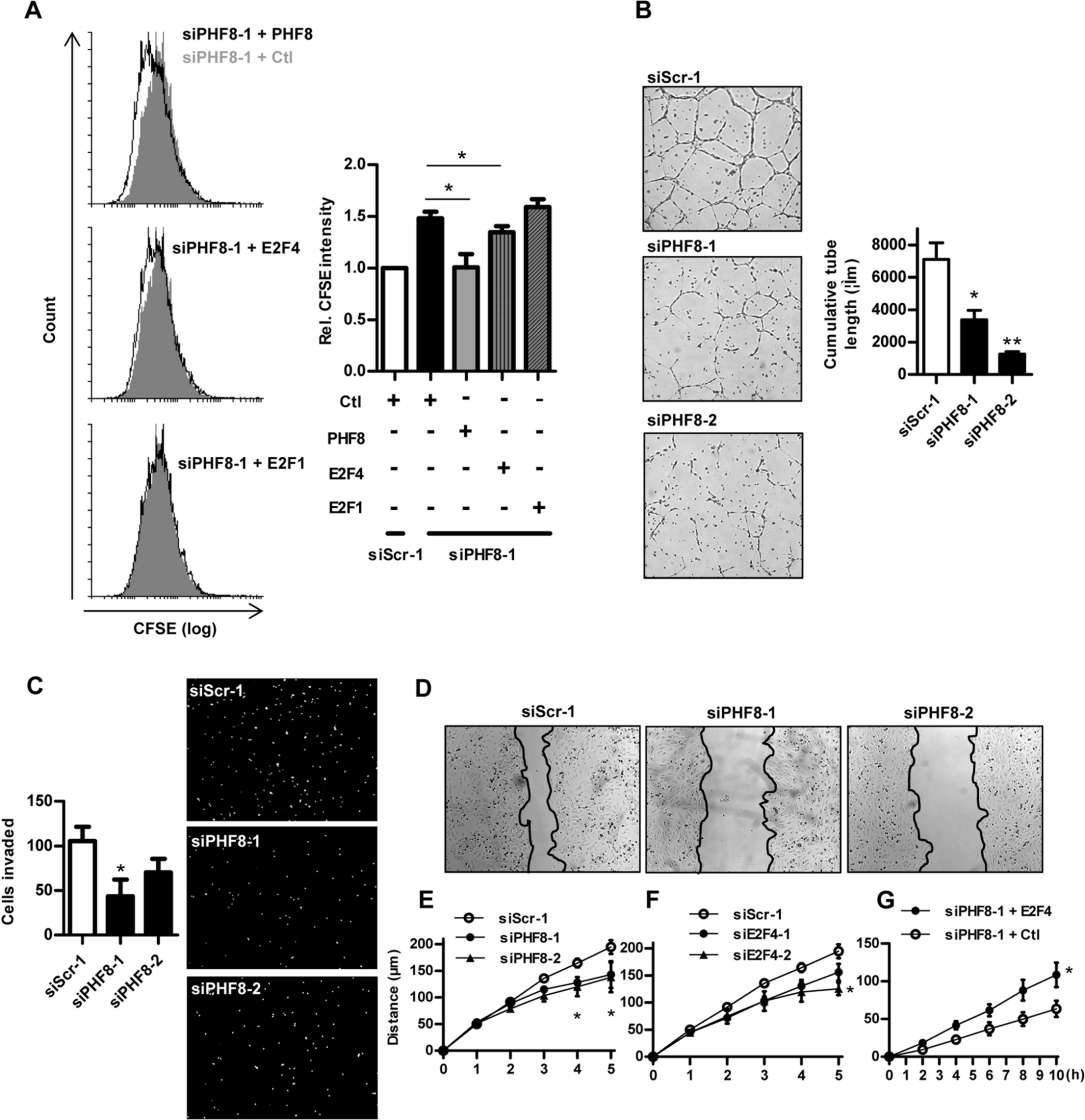
PHF8 maintains endothelial migration in an E2F4-dependent manner. A: Flow cytometry analyses of HUVEC proliferation with the CFSE dye after RNAi transfection against PHF8 with and without overexpression of PHF8 or E2F or E2F1 or DDK (FLAG tag) as control (Ctl) n = 5. Tube formation (B) Boyden chamber (C) and scratch wound assay (D&E&F&G) each with statistics of HUVECs transfected with control siRNA (siScr) or two different PHF8 siRNAs (siPHF8-1, siPHF8-2) or E2F4 siRNAs (siE2F4-1, siE2F4-2) with and without electroporation of plasmids coding for E2F4 and control (DDK (FLAG tag). n = 3. *p<0.05.

To exclude unspecific effects of the siRNA, rescue experiments for proliferation of HUVECs was performed. In PHF8-siRNA treated cells, overexpression of PHF8 restored the normal proliferation rate. Importantly, overexpression of E2F4 but not of E2F1 had a similar effect suggesting that loss of E2F4 expression significantly contributes to the effects of PHF8 siRNA **(Fig 5A)**. To determine the relevance of PHF8 in a more endothelial related context, tube formation assays were performed. The downregulation of PHF8 attenuated endothelial tube forming capacity **(Fig 5B).** Although this assay is a standard technique for angiogenesis research, the mechanisms affecting tube formation capacity are complex and not linked to endothelial cell proliferation or migration. The latter aspect was therefore studied separately, however, with a similar result: The knockdown of PHF8 decreased the migration of HUVECs in the Boyden chamber assay (**Fig 5C**) as well as in the scratch-wound assay **(Fig 5D&E)**. These assays were performed with a fairly short observation period (5 hours), making an effect of proliferation on the migration readout unlikely. Besides cell cycle progression, E2Fs have indeed additional effects [24–26] and thus may impact on migration. Indeed, also the depletion of E2F4 with two different siRNAs attenuated endothelial cell migration **(Fig 5F)**. Similar as for the proliferation experiments, overexpression of E2F4 could rescue normal endothelial migration after PHF8 knockdown **(Fig 5G)**. These observations suggest that a significant portion of the effects of PHF8 are mediated through the maintenance of E2F4 expression.

## Discussion

In this study we determined the role of PHF8 in endothelial cells. PHF8 facilitates endothelial cell proliferation and migration and this effect was probably a consequence of a PHF8-dependent demethylation of H3K9 at the E2F4 transcription start site.

The E2F family has an essential role in the control of cell cycle and there is growing evidence that PHF8 is a central regulator of cell cycle genes [16,27,28]. In the highly dedifferentiated epithelial HeLa cell line, PHF8 binds and modifies the H3K9 histone mark of the E2F1 promoter and increases transcription of E2F3 and E2F7 [16,27,28]. However, the targets of PHF8, if any, in primary mesenchymal cells like endothelial cells were unknown. Given that so far a function for PHF8 in humans was only attributed to neuronal tissue, it was an unexpected observation that this demethylase has an important function in the endothelium.

In this mesenchymal cell type our observations suggest that at least two different versions of PHF8 are expressed. SiRNA directed against different regions of the gene all resulted in a uniform loss of the expression of both proteins. This argues against but does not exclude that the two proteins represent splice variants of the PHF8 gene. Alternatively, the bands may reflect different post-translational modifications. Indeed, for epithelial cells it has been suggested that phosphorylation of PHF8 is required for its demethylase function [27].

Previous ChIP analysis in HeLa cells suggested that PHF8 locally reduces the abundance of the repressive H3K9me2 mark thus allowing transcription [27,29]. Our data, however, indicate that the activity of endogenous PHF8 is not very restricted but rather impacts on the global methylation state at least of H3K9me1 in endothelial cells. In contrast to PHF8 overexpression, depletion of PHF8 had no effect on global H3K9me2 and H4K20me1. This may points to a compensatory activation of other histone demethylases or reduced activity of methyltransferases. Alternatively, overexpression results in unspecific activity loss of targeting. Whether endogenous PHF8 affects specific H3K9me2 and H4K20me1 sites in HUVECs would require ChIP-seq experiments, which were not performed in the present study. Given that the H3K9me1/2 methylation mark is frequently enriched at repressive heterochromatin [30], our observations suggest that PHF8 acts as a rather global transcriptional activator.

Different to E2F1, E2F4 is thought to function as a gene repressor. It occupies promoters and reduces the expression of numerous genes involved in cell cycle control, DNA repair and apoptosis. This leads to a prolonged G1 progression and thus enables sufficient synthesis of proteins required for DNA replication and mitosis [31]. During cell proliferation, growth signals stimulate cyclin E/Cdk2 mediated phosphorylation of the retinoblastoma family proteins p107/p130. As one consequence E2F4 is released from its target promoters which results in S phase entry [32]. This model is compatible with our present observation: Knockdown of PHF8 reduced E2F4 expression and this was associated with premature S phase entry and a reduced G0/G1 state. The premature progression of G1 should result in problems with DNA replication and mitosis. Indeed, cells accumulated in S-phase and exhibited a prolonged G2 phase and apoptosis rates were increased after PHF8 knockdown. Alternatively, as a second potential mechanism of PHF8, altered H4K20 methylation was identified in the present study after overexpression of the protein. H4K20me1 impacts on cell cycle regulation as it supports chromatin condensation and mitotic progression [33].

It appears rather trivial that cell cycle arrest or dysfunction is associated with apoptosis [34,35]. E2F4 knockout mice exhibit an increased rate of cardiomyocyte apoptosis and this is mediated by several mechanisms. E2F4 directly blocks expression of the essential apoptosis-related genes E2F1, Apaf-1 and p73α through a mechanism involving the recruitment of histone deacetylase 1 for the induction of a repressive chromatin environment [22]. Our findings that PHF8 maintains E2F4 expression and that HUVECs devoid of PHF8 are more prone to undergo apoptosis are in agreement with the reduced E2F4 expression. It is, however, interesting to note that in endothelial cells PHF8 affects E2F4 but not E2F1 expression as previously reported for HeLa cells. E2F1 is considered to be a transcriptional activator and unlike E2F4, E2F1 induces apoptosis. The two proteins also differ in their oncogenic potential: Only E2F4 transgenic but not E2F1 transgenic mice developed skin tumors [36]. On this basis it is not surprising that also PHF8 is implicated in the development of several types of cancer [37].

As an endothelial cell specific readout the effect of PHF8 on cell migration and tube formation was determined. Our observations are consistent with previous reports in cancer cells in which depletion of PHF8 led to reduced migration and invasion of cancer cells [38–40]. PHF8 as a modifier of gene expression could regulate transcription of genes involved in migration and motility like cytoskeletal proteins, integrins and small GTPases of the Rho family. On the other hand, E2Fs are able to control expression of genes with functions other than cell cycle regulation [24]. Gene expression profiling of tumor-associated macrophages revealed that E2F3 for instance, controls gene expression which are associated with cytoskeleton rearrangements, cell migration and adhesion [41]. Furthermore, an involvement of E2Fs in neuronal migration has been reported and activation of E2Fs in smooth muscle cells promotes migration [25,26]. It is plausible that this mechanism is also important for vascular healing and the change from a quiescent to an active endothelial phenotype. The observation that depletion of E2F4 reduces endothelial wound closure supports this notion. It, however, remains to be determined whether this is a consequence of altered histone modification on specific endothelial target genes or indirectly mediated through E2F proteins.

Interestingly, the closest family member of PHF8, JHDM1D, which is also as a H3K9 demethylase, appears to have opposing effects to PHF8. It was reported that the enzyme inhibits tumor induced-angiogenesis and siRNA silencing did not affect cell proliferation or cell cycle progression [42]. The expression level of JHDM1D in the present study was however, greatly lower than that of PHF8. Interestingly, Osawa et al. reported that JHDM1D expression increased in mouse and human cancer cells under long-term nutrient starvation [42]. Therefore, additional work will be required to investigate whether JHDM1D is induced in endothelial cells under certain conditions and subsequently becomes functionally important.

Humans with mutation in the catalytic active JmjC domain of PHF8 suffer of mental retardation [10,11], but a vascular phenotype was not yet been reported. Thus, additional work will be needed to establish whether the effects observed in the present study in cultured cells are also operative in vivo.

In conclusion, the present work establishes PHF8 as a histone demethylase involved in endothelial cell phenotype control by maintaining E2F4 expression. Inhibition of PHF8 could be a potential novel anti-angiogenic strategy.
